# The Beauty and the Beast: pleural plaques due to asbestos exposure in a beauty salon worker. Who is guilty?

**DOI:** 10.47626/1679-4435-2024-1262

**Published:** 2025-01-07

**Authors:** Augusto Kreling Medeiros, Felipe Marques da Costa, Bruna Brandão Libânio, Pablo Rydz Pinheiro Santana, Ubiratan Paula Santos

**Affiliations:** 1 Radiology and Medical Imaging, Hospital Beneficência Portuguesa de São Paulo, São Paulo, SP, Brazil; 2 Pulmonology Team, Hospital Beneficência Portuguesa de São Paulo, São Paulo, SP, Brazil; 3 Pulmonology Division, Instituto do Coração, Hospital das Clínicas, Faculdade de Medicina, Universidade de São Paulo, São Paulo, SP, Brazil

**Keywords:** asbestos, talc, beauty and aesthetics centers, pleural diseases, barbering, amianto, talco, centros de embelezamento e estética, doenças pleurais, barbearia

## Abstract

This report highlights the occupational health risk of asbestos exposure in the hair and beauty salon industry, exemplified by the case of a 67-year-old hairdresser and manicurist. The patient’s medical history, work activities, and examination findings indicated potential asbestos-related pleural plaques. Occupational exposure to harmful chemicals, including asbestos in talc products, poses serious health risks. Despite asbestos bans in many countries, Brazil experienced extensive asbestos usage until 2012-2015, with a complete ban taking effect in 2023. This report emphasizes the need for ongoing discussions on asbestos detection and strict enforcement of bans, while ensuring asbestos-free talcum powders to protect the health of beauty industry workers.

## Abstract (pt)

Este relato destaca o risco à saúde ocupacional da exposição ao amianto no setor de salões de beleza, exemplificado pelo caso de uma cabeleireira e manicure de 67 anos. O histórico médico da paciente, suas atividades de trabalho e resultados de exames indicaram a possibilidade de placas pleurais associadas ao amianto. A exposição ocupacional a produtos químicos nocivos, incluindo o amianto presente em produtos de talco, representa graves riscos à saúde. Apesar da proibição do uso de amianto em muitos países, o Brasil utilizou amplamente esse material até 2012-2015, com uma proibição completa entrando em vigor em 2023. O relato enfatiza a necessidade de contínuas discussões sobre a detecção do amianto, a aplicação rigorosa das proibições e a garantia de produtos de talco sem amianto para proteger a saúde dos trabalhadores do setor da beleza.

## INTRODUCTION

This report sheds light on a major occupational health concern within the hair and beauty salon industry - occupational asbestos exposure. The case of a hairdresser and manicurist provides a compelling illustration of the potential risks faced by workers in this profession.

## CASE REPORT

This report outlines the case of a 67-year-old woman who sought medical attention for the evaluation of dyspnea and chest discomfort related to her regular activities. The patient’s medical history included chronic coronary artery disease, systemic arterial hypertension, and a previous smoking history of 35 pack-years. During examination, pulmonary auscultation revealed crackles predominantly in the right lung base. Peripheral oxygen saturation and blood pressure measurements were 98% on room air and 140/90 mm Hg, respectively. Further investigation with chest X-ray revealed a nodular opacity in the upper third of the right hemithorax, along with calcifications projected into the right diaphragm.

The woman’s profession involved working as a hairdresser and manicurist for approximately 32 years (1990-2022), 5 days a week, with 12-14-hour work shifts. At work, she reported handling talcum powder and wheat flour to produce a “plaster hair cap,” used for hair straightening, and the use of a hair dryer with hot air.

**Figure 1:**
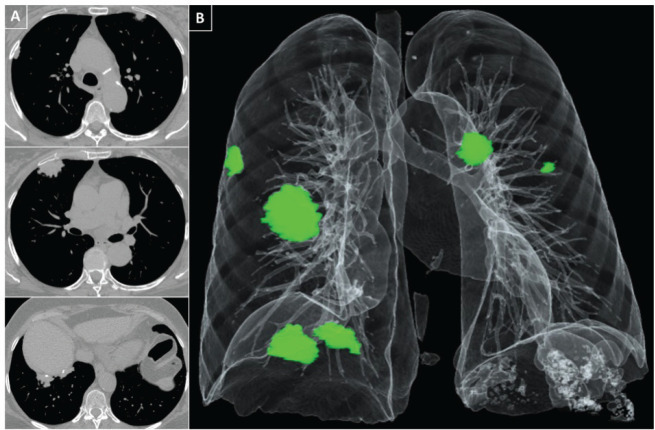
(A) Axial computed tomography (CT) slices in a craniocaudal sequence (from top to bottom) show bilateral pleural plaques. The plaques protrude against the lungs, exhibiting lobulated margins and discrete calcifications. (B) A 3D volume rendering highlights the distribution of the plaques in both hemithoraces, depicted in green.

Following the investigation, a chest computed tomography (CT) was remarkable for the presence of large bilateral pleural plaques, calcified on the right diaphragm; no parenchymal changes were observed (Figure 1). Pulmonary function tests (09/01/2022) showed forced vital capacity (FVC) of 2.52 L (100%), forced expiratory volume in 1 second (FEV1) of 2.07 L (102%), and FEV1/FVC ratio of 0.82; unresponsive to bronchodilators.

Based on the whole clinical scenario, a presumptive diagnosis of pleural plaques related to asbestos exposure was made. The patient was informed of the nature of pleural plaques and their association with asbestos exposure. She will continue to attend regular follow-up appointments to monitor her clinical, functional, and radiological status. She was advised of the importance of avoiding any further asbestos exposure.

## DISCUSSION

Occupational exposure to a wide range of chemicals is a prevalent concern across various industries, including beauty and personal care. Workers in nail, hair, and beauty salons often encounter numerous potentially harmful chemical products, such as toluene, formaldehyde, and dibutyl phthalate, found in nail polish and other cosmetic products, which can adversely affect health over time.^1^

In the case reported here, the hypothesis of pleural plaques resulting from asbestos exposure was based on the well-documented contamination of talc with this hazardous fiber. This contamination poses not only a risk of non-malignant diseases but also a grave concern for the development of mesothelioma.^2,3^ Furthermore, the use of asbestos in the coatings of hair dryers, which were part of the patient’s daily work routine, adds to the potential exposure. During a significant portion of the patient’s working life, the use of asbestos products remained common in Brazil, and the quality control of cosmetic talc is often insufficient to detect possible contamination. Although exposure may not have been intense enough to cause asbestosis, it was sufficient to induce pleural disease. This highlights an important aspect of asbestos-related health conditions: both malignant and non-malignant pleural diseases are more strongly associated with latency, the time elapsed since exposure, than with the amount of asbestos inhaled.^4^

Regarding her occupation, it is essential to acknowledge that certain products utilized in nail and hair salons might contain asbestos fibers, which can be released into the air during product application or removal. Until the 1970s, asbestos was used as a heat insulator in some hair dryers, potentially exposing workers to asbestos fibers. However, it is crucial to note that, since the 1970s, many countries have banned the use of asbestos in hair dryers, and modern hair dryers are typically asbestos-free.

As of March 2019, a total of 66 nations banned asbestos, and an additional 10 nations are placing restrictions on asbestos use. While all 28 European Union countries have banned the use of asbestos, this toxic mineral remains legal in the United States.^5^ Despite the bans and restrictions, Brazil experienced significant asbestos product usage until 2012-2015, with a comprehensive ban on asbestos, from extraction to commercialization, becoming effective only in 2023. Considering the period of occupational exposure in our patient’s case, it is possible but unlikely that hair dryers could directly serve as a source of asbestos exposure.

On the other hand, talc-containing products, such as cosmetic powders and body powders, may carry a risk of asbestos fiber contamination due to the presence of naturally occurring asbestos (NOA) in talc mines.^6^ Of particular concern is our patient’s involvement in the manipulation of cornstarch hair masks, commonly known as *touca de gesso* in Brazil, used as a dry shampoo. These masks consist of a blend of cornstarch and various other ingredients, which may include talc powder, as in the present case. The well-known association between asbestos in talc used for cosmetics and the development of mesothelioma and other health issues has led to high-profile lawsuits against talc manufacturers and distributors.^7^

While some sources in the medical literature present controversial data regarding the presence of asbestos in talc products, many others continue to raise concerns about the safety of such products.^8-11^ In the 2023 article by Moline et al.,^12^ significant evidence is provided to reinforce these concerns: the study reported a case series involving 166 individuals with substantial exposure to asbestos-containing cosmetic talc products who subsequently developed mesothelioma. In 44 cases, potential or documented alternate exposures, apart from cosmetic talc, were present, while in 122 cases, no other source of asbestos exposure was identified until the history of asbestos-containing cosmetic talc use was elicited. Among the 166 individuals, 4 hairdressers, all with more than 25 years of talcum powder usage, were affected, with 2 of them being potentially exposed through alternate sources such as hair dryers or automotive friction materials. Furthermore, several other case series have also identified hairdressers and barbers with occupational exposure to cosmetic talc who developed mesothelioma.^13,14^

In conclusion, this case report serves as a stark warning for workers in nail, hair, and beauty salons who may be exposed to hazardous chemicals and materials. The presence of pleural plaques in our patient highlights a significant risk marker for lung cancer and mesothelioma associated with asbestos exposure. The nature of the patient’s occupational activity, often conducted in confined environments with poor ventilation and long working hours over many years, may explain the potential cumulative exposure to asbestos present in talc cosmetics, even in seemingly small amounts.

Given the risks to which these workers are exposed, this report emphasizes the urgent need for ongoing discussions on asbestos detection and strict enforcement of asbestos bans, as already mandated by law. Additionally, it underscores the importance of ensuring that talcum powders, whether used for cosmetic purposes or not, are free of asbestos. By raising awareness of these issues, we can work towards safeguarding the health and well-being of individuals working in the beauty industry.
